# Combination of computational techniques and RNAi reveal targets in *Anopheles gambiae* for malaria vector control

**DOI:** 10.1371/journal.pone.0305207

**Published:** 2024-07-05

**Authors:** Eunice O. Adedeji, Thomas Beder, Claudia Damiani, Alessia Cappelli, Anastasia Accoti, Sofia Tapanelli, Olubanke O. Ogunlana, Segun Fatumo, Guido Favia, Rainer Koenig, Ezekiel Adebiyi

**Affiliations:** 1 Covenant University Bioinformatics Research (CUBRe), Covenant University, Ota, Ogun State, Nigeria; 2 Department of Biochemistry, Covenant University, Ota, Ogun State, Nigeria; 3 School of Biosciences & Veterinary Medicine, University of Camerino, Camerino, Italy; 4 Department of Biology, University of York, York, United Kingdom; 5 Medical Department II, Hematology and Oncology, University Medical Center Schleswig-Holstein, Kiel, Germany; 6 University Cancer Center Schleswig-Holstein, University Medical Center Schleswig-Holstein, Kiel and Lübeck, Germany; 7 Institute for Infectious Diseases and Infection Control (IIMK, RG Systemsbiology), Jena University Hospital, Jena, Germany; 8 Department of Life Sciences, Imperial College, London, United Kingdom; 9 African Center of Excellence in Bioinformatics & Data Intensive Science, Makerere University, Kampala, Uganda; 10 Department of Non-Communicable Disease Epidemiology, London School of Hygiene & Tropical Medicine, London, United Kingdom; 11 Division of Applied Bioinformatics, German Cancer Research Center (DKFZ), Heidelberg, Germany; ICMR-National Institute of Malaria Research, INDIA

## Abstract

Increasing reports of insecticide resistance continue to hamper the gains of vector control strategies in curbing malaria transmission. This makes identifying new insecticide targets or alternative vector control strategies necessary. CLassifier of Essentiality AcRoss EukaRyote (CLEARER), a leave-one-organism-out cross-validation machine learning classifier for essential genes, was used to predict essential genes in *Anopheles gambiae* and selected predicted genes experimentally validated. The CLEARER algorithm was trained on six model organisms: *Caenorhabditis elegans*, *Drosophila melanogaster*, *Homo sapiens*, *Mus musculus*, *Saccharomyces cerevisiae* and *Schizosaccharomyces pombe*, and employed to identify essential genes in *An*. *gambiae*. Of the 10,426 genes in *An*. *gambiae*, 1,946 genes (18.7%) were predicted to be Cellular Essential Genes (CEGs), 1716 (16.5%) to be Organism Essential Genes (OEGs), and 852 genes (8.2%) to be essential as both OEGs and CEGs. RNA interference (RNAi) was used to validate the top three highly expressed non-ribosomal predictions as probable vector control targets, by determining the effect of these genes on the survival of *An*. *gambiae* G3 mosquitoes. In addition, the effect of knockdown of arginase (AGAP008783) on *Plasmodium berghei* infection in mosquitoes was evaluated, an enzyme we computationally inferred earlier to be essential based on chokepoint analysis. Arginase and the top three genes, AGAP007406 (Elongation factor 1-alpha, Elf1), AGAP002076 (Heat shock 70kDa protein 1/8, HSP), AGAP009441 (Elongation factor 2, Elf2), had knockdown efficiencies of 91%, 75%, 63%, and 61%, respectively. While knockdown of HSP or Elf2 significantly reduced longevity of the mosquitoes (p<0.0001) compared to control groups, Elf1 or arginase knockdown had no effect on survival. However, arginase knockdown significantly reduced *P*. *berghei* oocytes counts in the midgut of mosquitoes when compared to LacZ-injected controls. The study reveals HSP and Elf2 as important contributors to mosquito survival and arginase as important for parasite development, hence placing them as possible targets for vector control.

## Introduction

Vector control interventions remain potent strategies for controlling the transmission of malaria, a disease that remains a global menace [1]. These interventions include larval control methods, use of insecticide-treated nets and indoor residual spraying [2]. However, these vector control strategies are approaching the limit of their effectiveness [3], resulting in the consistently high morbidity rates reported annually [1]. Consequently, there is an urgent need to introduce innovative vector control strategies. Other vector control interventions such as RNA interference (RNAi) based biopesticides, generation of refractory mosquitoes, sterile insect techniques are currently gaining attention. However, these techniques are dependent on the identification of appropriate targets. An important tool in functional genomics which can be used to investigate and characterize promising targets is RNAi, an important gene silencing technique that provides insight into the function of a gene and can be successfully applied to investigate gene knockdown in mosquitoes [4]. Using this technique, the synthesis of proteins that play a role in the survival, fecundity, metabolism, vectorial capacity, and the behaviour of mosquitoes and insects generally, have been suppressed, thus, unveiling some of these proteins as possible targets for vector control. For example, RNAi provided insight on the role of TEP1, LRIM1, and APL1 as crucial genes for the immune response in *Anopheles* [5, 6]. Similarly, several proteins involved in insecticide resistance, transport of molecules, reproductive fitness, and host-seeking behaviour have been identified through RNAi. Examples include ABCG4 [7], CYP 450s [8], c-Jun N-terminal kinase (JNK) pathway components [9], Aquaporin 3 [10], and G protein-coupled receptors (GPCRs), which play a role in development, visual, gustatory and olfactory sensing, homeostasis, and hormonal regulation [11].

RNAi has been described to be a promising technique for pest and mosquito control [12–16]. It is an important tool for identifying potential insecticidal targets that could be explored to develop insecticides or other products to limit the burden of mosquitoes on human health [17]. The accuracy and specificity of the technique make RNA-based biopesticides to be considered as alternatives to chemical-based insecticides [18–20]. Since they are specific, unwanted effects could be reduced and thus having reduced or no effects on non-target organisms. Some targets which have been investigated as RNAi biopesticides for vector control include chitin synthase [21] and 3-hydroxykynurenine transaminase [17, 22]. Likewise interfering RNA pesticide (IRP) corresponding to the mosquito *Shaker* and GPCR *dopamine 1 receptor (dop1)* genes have been tested and found to have adulticide and larvicidal activities against different mosquito species [23, 24]. However, employing experimental techniques to screen every gene in a disease vector to identify potential targets is a tall order.

In turn, computational methods can be employed to predict essential proteins in organisms [25] Essential genes are considered to be crucial for the survival or reproductive success of an organism [26, 27]. These essential genes in disease vectors could serve as possible vector control targets. Employing computational techniques can lead to refined smaller lists of genes, which could then be validated by experimental techniques to determine their suitability for vector control. Computational techniques for predicting essential genes range from simple algorithms like chokepoint analysis in metabolic networks to more complex techniques like machine learning. Chokepoint analysis has been employed to identify possible insecticidal targets in *Anopheles gambiae*, a major malaria vector [28]. An example of a gene predicted as essential in *An*. *gambiae* using the chokepoint criteria is arginase, which was observed to be highly expressed in the midguts of *Plasmodium berghei* infected mosquitoes compared to their blood-fed counterparts [29]. Hence, arginase could contribute to the development of the parasite in the mosquito and could possibly serve as a target for vector control. However, experimental validation of these predictions remains to be done. Beder *et al*. [30] developed a machine learning based-technique trained on six model organisms to predict essential genes using a combination of leave one organism out cross-validation and orthology based approaches. In this present study, we (i) experimentally followed up on the computational prediction of arginase to be essential, and (ii) applied the machine learning method to predict essential genes in *An*. *gambiae*, and experimentally validated a selected short list of the predicted genes using the RNAi knockdown technique.

## Methods

### The machine learning method to identify essential genes *in silico*

We applied a modification of the CLassifier of Essentiality AcRoss EukaRyote which is a machine learning classifier for essential genes which was trained on the six model organisms: *Caenorhabditis elegans*, *Drosophila melanogaster*, *Homo sapiens*, *Mus musculus*, *Saccharomyces cerevisiae and Schizosaccharomyces pombe* [30]. The machine was trained on 60,381 genes, using 41,635 features based on seven different sources including protein and gene sequence, functional domains, topological features, evolution/conservation, subcellular localization, and gene sets from Gene Ontology.

#### Feature generation

Essential genes for *An*. *gambiae* were predicted using the same features used for the model organisms. The *An*. *gambiae* str. PEST genome (GenBank assembly accession: GCA_000005575.1) was used to generate the gene and protein sequence features. The tools seqinR [31], protr [32], CodonW (http://codonw.sourceforge.net/) and rDNAse [33] were used to calculate protein and gene sequence features. For genes with isoforms, the features were generated individually for each isoform and the median of all was calculated. seqinR provided simple protein sequence information including the number of residues, the percentage of physico-chemical classes and the theoretical isoelectric point. Most protein sequence features were obtained using protr comprising autocorrelation, conjoint triad, quasi-sequence order and pseudo amino acid composition. CodonW was used to calculate simple gene descriptors like length and GC content, frequency of optimal codons and the effective number of codons. rDNAse provided DNA descriptors such as auto covariance, pseudo nucleotide composition, and kmer frequencies (*n* = 2–7). Domain features were calculated using the tools from the Technical University of Denmark (http://www.cbs.dtu.dk/services/), comprising the prediction of membrane helices and beta-turns, cofactor binding, acetylation and glycosylation sites. Topology features were derived from protein-protein-associations (PPA) using the STRING v11 [34] database. These features comprised of degree, degree distribution, betweenness, closeness and clustering coefficient using the Python library NetworkX. Conservation features were calculated by the number of homologous proteins of a query protein in the complete RefSeq database [35] using PSI-BLAST [36]. As features, the number of proteins identified with *e*-value cutoffs from 1e−5 to 1e-100 (in 1e−5 multiplication steps) were used. An alignment coverage score (ACS) was calculated for hits with a cutoff ≤1e−30 as we described formerly [37]. Furthermore, the number of homologous sequences with a score from 0 to 0.95 in 0.05 steps were calculated. Similarly, the number of paralogous sequences were calculated. Here, blastn alignment results with an *e*-value cutoff ≤1e−30 were used as input for the score. Subcellular localization features were predicted by the tool DeepLoc [38]. DeepLoc assigns a score for each protein to its localization in 11 eukaryotic cell compartments. Gene set features were derived from all Gene Ontology (GO) terms present in all analyzed organisms, similar to Chen *et al*. [39]. Here, not only the characterization of the query gene was taken into account, but also of its neighbors in the protein association network. By this, the features were more robust against false gene set annotations. The neighbors of the query gene were assembled employing the gene network definitions of STRING v11. A Fisher’s exact test for enrichment of interaction partners was performed for each of the gene sets. The log 10 values of the *P*-values were used as features.

#### Defining the gold standard

Essentiality information was derived from the six species: *C*. *elegans*, *D*. *melanogaster*, *H*. *sapiens*, *M*. *musculus*, *S*. *cerevisiae* and *S*. *pombe*. For *D*. *melanogaster*, *H*. *sapiens* and *M*. *musculus*, screening data was collected from screens of cellular essential genes (CEG) and organismal essential genes (OEG). For *C*. *elegans*, and the yeasts only CEG screening data was available. This essentiality information was derived from Online GEne Essentiality (OGEE) [40] and Database of Essential Genes (DEG) [41] databases and the literature (for more details see [30]). For genes with different essentially status in different screens, a majority voting was performed. For human cell line screens, a gene had to be studied in at least five experiments as described formerly by Guo *et al*. [42].

#### Normalization, feature reduction and machine learning

Data analysis was performed using R. Values of each feature were z-transformed and each value was rounded to deciles. For feature selection and learning, the data was randomly split into training (80%) and testing (20% of the data). Using the training set, feature selection was performed in two steps: first, LASSO was applied using the glmnet package [43] (cv.glmnet function, alpha = 1, type.measure = ‘auc’). In the second step, collinearity was reduced by removing highly correlating features with Pearson correlation coefficients *r* ≥ 0.70. Next, class imbalances were addressed during training using SMOTE [44]. The classifiers were trained using Random Forest (RF) from the caret [45] package. For RF, *tuneLength* in the *train* function was set to 3 resulting in three predictors randomly sampled at each split. For each organism, a stratified randomized 5-fold cross-validation was performed in which feature selection, parameter tuning and training of the classifiers was done using 80% of the data. 20% of the data was used for testing the performance. Leave-one-organism-out cross-validation: For each individual species (five species for CEG, four for OEG predictions), five machines were trained. Essential genes for the left-out species were predicted with machines trained on the according CEG or OEG data sets of the other organisms. Thereby the classifiers for each (non-left out) species provided an essentiality prediction score between zero and one and the average of these scores was used for the prediction of a gene to be essential in the left-out species. TPM values from an RNA-seq dataset (E-MTAB-9241) were used to aid selection of predicted genes for the experimental phase [46, 47].

### Experimental methods

#### Mosquito handling

*An*. *gambiae* G3 strain was maintained at 27–29°C and 75–90% relative humidity with a 12:12 light-dark photoperiod. Adults were provided with 5% sucrose solution *ad libitum* [48]. For reproduction, female mosquitoes were fed on BALB/c mice for 30 min. Egg dishes were placed in cages 48 h post-blood-feeding and retrieved 24 h later. Mosquitoes were reared according to MR4 protocol [49]. Eggs were bleached with 1% bleach and rinsed three times with 0.05 g/L salted deionized water upon egg collections. Bleached eggs were hatched on the following day in a tray containing 500 mL of 0.05g/L salted deionized water and larva food to a final concentration of 0.02%. Splitting of L1 larvae was carried out either in the evening of the day of hatching or the day after. Larvae were split into trays, with each tray containing approximately 250 larvae in 500 mL of 0.05 g/L salted deionized water and larva food to a final concentration of 0.02%. A volume of food (3–10 mL) was added to the tray daily depending on the larvae stage. Larvae food (50 g) comprised a mixture of tuna fish meal (20g), liver powder (20g), and vitamin mix (10g). An aliquot of larvae food (1 g) was dissolved in 50 mL of water (yielding 2%), and appropriate volumes were taken from this. Upon pupation, pupae were collected into cups having clean water and placed in a cage to allow the emergence of adults.

#### Mice handling

BALB/c mice were used as *P*. *berghei* vertebrate hosts and in blood-feeding mosquitoes for reproduction. The mice were maintained in the animal facilities of the University of Camerino, Camerino, Italy. All animal rearing and handling was carried out according to the Italian Legislative Decree (116 of 10/27/92) on the “use and protection of laboratory animals” and in agreement with the European Directive 2010/63/UE. The experimentation was approved by the Ethical Committee of University of Camerino. According to the method of Cappelli et al. [50], BALB/c mice were maintained at 24°C, fed on standard laboratory mice pellets (Mucedola S.r.l., Milano, Italy) and provided with tap water *ad libitum*. Mice were anesthetized using a mixture of 10 mg/mL prequillan (ATI-srl), 20 mg/mL sedaxylan (Dechra) and 1X Phosphate-buffered saline (PBS). Each mouse was injected with 0.1 mL of this mixture intraperitoneally and used to feed mosquitoes 15 mins after injection.

#### Primers

Primers with T7 tail for dsRNA synthesis were designed using the E-RNAi website (https://www.dkfz.de/signaling/e-rnai3/). All qPCR primers were designed using primer wizard in Benchling (www.benchling.com), which is powered by Primer3 (https://primer3.org/). Care was taken to ensure that designed primers were target gene specific. Primers were synthesized by Metabion (www.metabion.com). All primers used in this study are provided in S1 Table.

#### RNA interference

Total RNA extraction from a pool of five whole mosquitoes was carried out using RNAzol RT (Sigma) according to the manufacturer’s instructions. Complementary DNA (cDNA, 10 μL) was synthesized from 500 ng total RNA using PrimeScript RT reagent kit (Takara Bio) according to the manufacturer’s instruction and incubated at 37 ℃ for 15 min, then 5 ℃ for 5 s. A fragment from each target gene was amplified using cDNA and target specific primers having the T7 promoter tag sequence TAATACGACTCACTATAGGG incorporated into their 5’ end (to enable *in vitro* transcription by T7 polymerase). PCR fragment of LacZ served as a control and was synthetized from *E*. *coli* expressing LacZ using LacZ specific primers. All primers used are provided in S1 Table. An aliquot of this PCR reaction was used as a template for dsRNA synthesis *in vitro* using TranscriptAid T7 High Yield Transcription Kit (Thermo Scientific) and purified following the manufacturer’s instruction. DNA was removed by DNase I digestion, while all proteins and free nucleotides were removed by phenol chloroform extraction according to the manufacturer’s protocol. dsRNA was eluted with DEPC treated water and its concentration measured using Nanodrop 1000 spectrometer (Thermo Fisher Scientific, USA). Gel electrophoresis (1% TBE agarose) was performed on an aliquot of the PCR products to confirm that products of the expected size were synthesized for each gene. Similarly, aliquots of the synthesized dsRNA were evaluated on a 2% TBE agarose gel.

#### Mosquito injection

Two to three-day-old female mosquitoes were anaesthetized on ice and injected with 138 nL (5 μg/μL) target-specific dsRNA or LacZ control dsRNA using a Drummond Nanoject II Automatic Nanoliter Injector (3-000-205-A, Drummond Scientific, Broomall, PA, USA) and glass microcapillary injection needles according to the method described by Mancini *et al*. [48]. The microcapillary injection needles used were obtained by using Flaming/Brown Micropipette Puller System Model P-1000 (Sutter Instruments Company, Novato, CA, USA) to pull glass capillaries (BF150-86-10, Sutter Instruments, Novato, CA, USA). For survival experiments, anesthetized mosquitoes injected with 1X PBS were used as a handling control. Mosquitoes not subjected to injection, but had undergone cold anesthetization, were used as an untreated control. Mosquitoes which died within 24 hours post-injection were discarded from the analysis. Fifteen mosquitoes were injected to determine knockdown efficiency by quantitative-PCR (qPCR) analysis (5 mosquitoes per replicate, 3 replicates), while 70 female mosquitoes per treatment were used for longevity analysis (70 mosquitoes per replicate, 4 replicates). For *P*. *berghei* infection, 200 female mosquitoes per treatment were injected. After injections, mosquitoes were maintained in the insectary under standard conditions.

#### Real-time quantitative PCR (RT-qPCR)

RNA was extracted from whole mosquito samples at intervals of 24 h post-dsRNA injection– 24 h, 48 h, 72 h and 96 h for the arginase experiment. For the experiments of HSP, Elf2 and Elf1, RNA was extracted from whole mosquito samples three days post-dsRNA injection. RNA was reverse-transcribed into cDNA using iScript™ gDNA Clear cDNA Synthesis Kit (BioRad). Real-time quantitative PCR was conducted using 4 μL of HOT FIREPol® EvaGreen® qPCR Supermix (Solis Biodyne, Estonia), 2 μL of template (cDNA), 2.5 μL of 1 μM Primer mix (combined forward and reverse primers) which was made up to a final volume of 20 μL using nuclease-free water. PCR amplification was performed by preheating the reaction to 95°C for 12 min, followed by 40 PCR cycles (95°C for 30 s, 60˚C for 30 s, and 74˚C for 30 s), a melt curve run from 65–95 ℃, with an increase in temperature by 0.5 ℃/cycle every 5 s. *An*. *gambiae* ribosomal S7 gene was used as an internal reference gene for the normalization of each target gene, and the results were normalized further against the LacZ injected control group. Relative expression levels of the genes were reported as 2-ΔΔCT [51]. The amplification efficiency of all qPCR primers used was determined.

#### Survival assay

Longevity was assessed to determine whether knockdown of HSP, Elf2 or Elf1 affected *An*. *gambiae* survival. Longevity was evaluated using a total of 280 female mosquitoes per treatment (70 mosquitoes per replicate, 4 replicates). The rate of survival was monitored until 100% mortality was reached for all six treatments (HSP dsRNA, Elf2 dsRNA, Elf1 dsRNA, LacZ dsRNA, PBS, and not injected).

For arginase knockdown, longevity was assessed using a total of 140 female mosquitoes per treatment (70 mosquitoes per replicate, 2 replicates). The setup included three treatment groups–Arg dsRNA, LacZ dsRNA, and the not injected treatment groups. All mosquitoes, including those in the not injected treatment groups, were exposed to a naïve blood meal from mice 48 h post-dsRNA injection. A blood meal was introduced because an effect of knockdown of these genes on *P*. *berghei* development was hypothesized. The rate of survival was monitored until 19 days post-blood meal. Survival analyses were performed using the Kaplan–Meier method [52, 53] and significance between groups determined by Log-rank (Mantel-Cox) tests. Graphs were plotted using the ggsurvplot function in the R-programming software [54].

#### *P*. *berghei* infection

The murine malaria parasite, *P*. *berghei* was used in this study as a model organism for investigation of human malaria. Three mice were infected with *P*. *berghei* (GFP_CON,_ PbGFP_CON_) from frozen capillary stocks diluted in 200 μL of PBS (7.2 pH) through intraperitoneal injection. The level of parasitemia in mice was determined four days after infection using slides with methanol fixation of air-dried blood smears taken from the tail of the mice, followed by staining with 15% (w/v) Giemsa solution. Parasitemia was counted under an optical microscope (Olympus CX21). The mouse with the best parasitemia was selected as the donor mouse for passaging to recipient mice. Infection of recipient mice was carried out according to the method of Cappelli *et al*. [50] with slight modification. Eight-week-old female mice (18–25 g) were infected with *P*. *berghei* directly by an intraperitoneal injection of 5 x 10^6^ infected erythrocytes (from a donor mouse) diluted in 200 μL of PBS (7.2 pH). Parasitemia and gametocytemia were determined in a 15% Giemsa-stained blood smear obtained from recipient infected mice three days after infection using an optical microscope. Recipient mice were used in feeding mosquitoes when parasitemia was between 8 and 11%, and gametocytemia was between 1.2 and 3.8. Prior to feeding of mosquitoes with mice, mice were anesthetized using a mixture of 10 mg/mL prequillan, 20 mg/mL sedaxylan and 1X PBS, the mice were used to feed mosquitoes 15 mins after being anesthetized.

Non-fed and partially fed females were removed from the cage. To allow parasite development, mosquitoes were kept at 19 ℃ and 70% humidity. Midguts from a total of 50 mosquitoes per treatment group were dissected 10 days post-blood meal (PBM) to confirm the presence of oocysts using fluorescent microscopy. The number of oocytes in each midgut was counted and compared to the LacZ control.

### Statistical analysis

Statistical analysis on gene expression data and oocytes count data were performed at a 95% confidence interval in GraphPad Prism 5. Relative gene expression data were presented as mean ± SEM, and statistical significance was determined by one-way analysis of variance (when comparing more than two groups) and Bonferroni post hoc test. Where only two groups were compared, the paired T-test was used. Data from survival analysis were analyzed using the Kaplan–Meier method [52], presented as mean ± confidence interval, and a Log-rank test was used to determine whether survival curves were statistically significant between the target-specific dsRNA treated groups and control groups. Survival curves were plotted using R software. Oocytes count data from infection experiments was presented as median on a dot plot, with each dot representing the number of oocytes/midgut. Statistical significance was between oocytes count from LacZ and the treatment group was determined by a Mann Whitney test.

## Results

### Machine learning results

**Fig 1:**
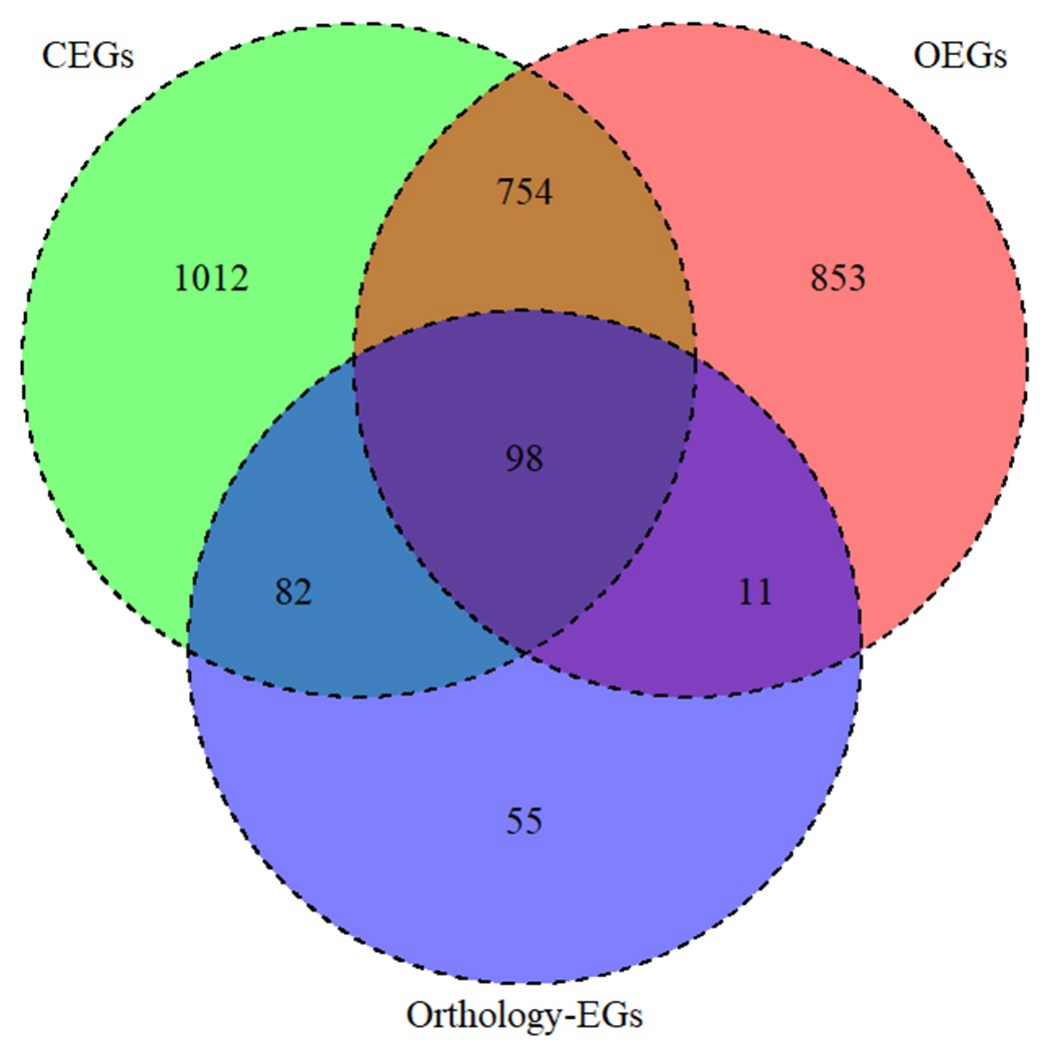
Venn diagram of the number of predicted essential *An*. *gambiae* genes by CLEARER and orthology algorithm. CEGs: Cellular essential genes, OEGs: Organism essential genes, Orthology-EGs: Orthology essential genes.

Predictions were carried on a total of 10426 genes in *An*. *gambiae*. Using the CLEARER approach, 1946 genes (18.7%) were predicted to be CEGs, 1716 (16.5%) to be OEGs and 852 genes (8.2%) to be essential as both OEGs and CEGs. Using the orthology based approach, only 249 (2.4%) genes were predicted to be essential. Combining both CLEARER and orthology based approach, only 94 genes (0.9%) were predicted to be essential (Fig 1). The results of the predictions for all 10426 genes in *An*. *gambiae* are provided in S1 File.

Some of the genes predicted as essential based on the three approaches had very low prediction scores based on the CLEARER approach (<0.2). To proceed further, we focused on the essentiality scores from CLEARER, selecting the top 250 ranked genes, which belonged to the top 2.5% of the scores. We proposed that an essential gene in addition to being essential in our predictions, should be highly expressed. Only thirteen of the genes belonging to the top 2.5% had high gene expression values (i.e., mean TPM > 1000), suggesting they are highly expressed in all conditions. The median, 25th and 75^th^ percentile TPM values of all genes are provided in S1 File. We experimentally validated the top 3 highly expressed predicted genes which were non-ribosomal genes. These genes are presented in Table 1.

**Table 1:** Selected predicted genes for experimental validation.

Gene	AGAP007406	AGAP002076	AGAP009441
**Uniprot annotation**	Q7PT29 (Elongation factor 1-alpha)	A7UVK8 (Heat shock 70kDa protein 1/8)	Q7PTN2 (Elongation factor 2)
**Essentiality score**	0.38759	0.26499	0.60084
**CE prediction**	Essential	Essential	Essential
**OE prediction**	Essential	Essential	Essential
**Essential orthologs**	6	14	14
**Non-essential orthologs**	11	52	6
**Orthology Prediction**	Non-essential	Non-essential	Essential
**TPM (Mean)**	7919.616	3462.944	3025.191
**TPM (Median)**	8114.321	3602.958	3051.977
**TPM (25th Percentile)**	6072.29	2342.043	2166.012
**TPM (75 Percentile)**	10231.74	4560.174	4026.935
***Drosophila melanogaster* Ortholog**	FBgn0000557	FBgn0266599	FBgn0000559
**OGEE Essentiality of *Drosophila melanogaster* Ortholog**	Conditionally essential	Conditionally essential	Essential

Essentiality score is based on CLEARER prediction; Essential/Non-essential orthologs are based on OGEE and DEG database; CE prediction: Cellular essentiality prediction; OE prediction: Organism essentiality prediction.

While AGAP009441 was predicted to be essential by all three methods (belonging to one of the 98 genes in Fig 1), AGAP007406 and AGAP002076 were predicted to be essential as both CEGs and OEGs, but not essential by orthology based approach (thus, belonging to the 754 genes in Fig 1). However, both genes had 14/66 (21%) and 6/17 (35%) essential orthologs, respectively and their *D*. *melanogaster* ortholog are conditionally essential based on OGEE Essentiality.

### Effect of knockdown of arginase on survival of mosquitoes and *P*. *berghei* development

**Fig 2:**
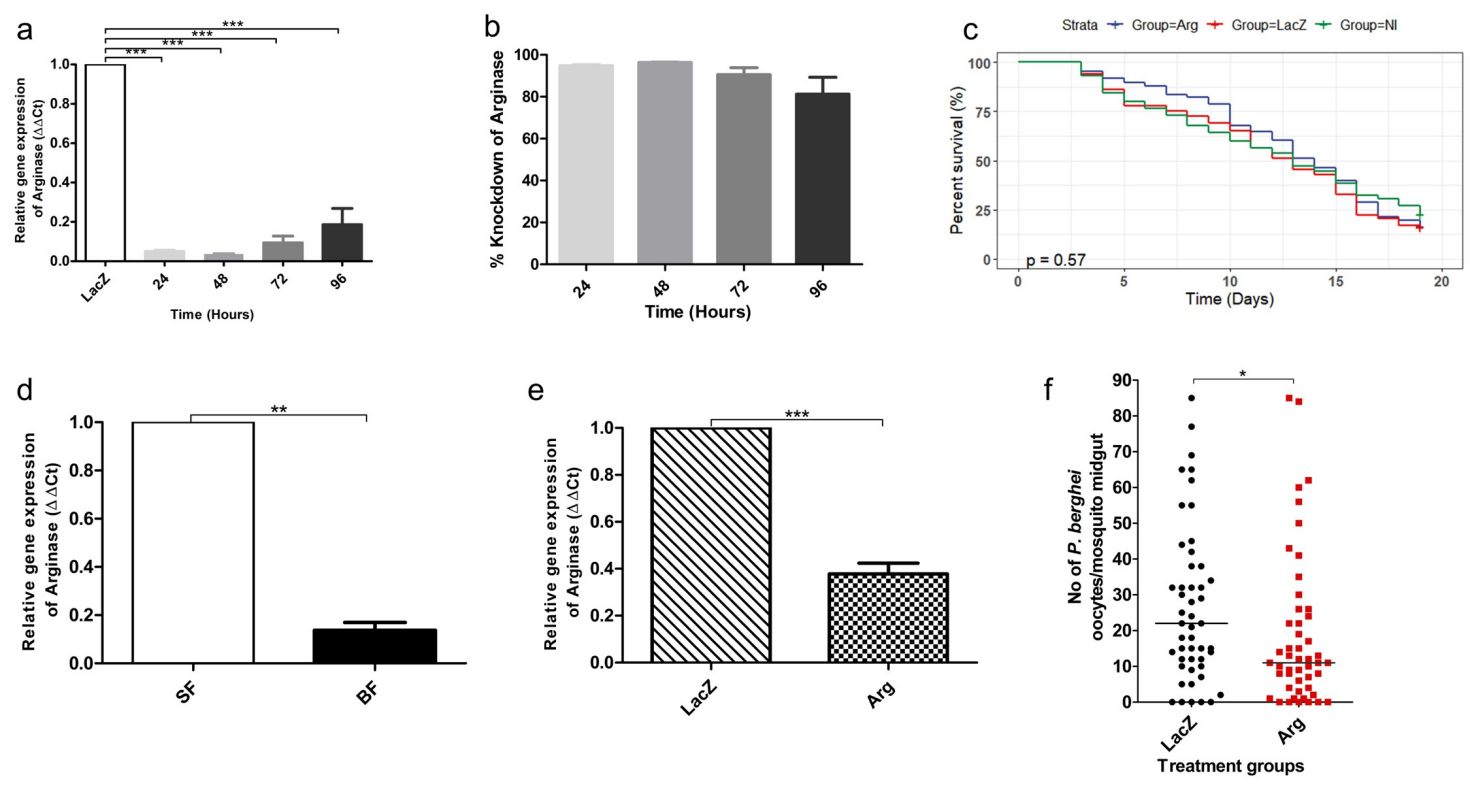
(a) Relative gene expression of arginase at 24, 48, 72 and 96 h after dsRNA injection in *An*. *gambiae* G3 mosquitoes (n = 6) (b) Percentage knockdown of arginase at 24, 48, 72 and 96 h after dsRNA injection in *An*. *gambiae* G3 mosquitoes (n = 6) (c) Percentage survival of female *An*. *gambiae* blood-fed at 48H after knockdown of arginase (d) Relative gene expression of arginase in not injected blood-fed (BF) *An*. *gambiae* G3 compared to not injected sugar-fed (SF) 24 h after blood feeding (n = 3) (e) Relative gene expression of arginase in Blood fed (BF) *An*. *gambiae* G3 mosquitoes injected with LacZ dsRNA or Arg dsRNA at 72 h post dsRNA injection (n = 5) (Blood feeding carried out 48 h after injection) (f) *P*. *berghei* oocytes counts 10 days post infection in female *An*. *gambiae* fed with blood of parasitized mice 48 h after knockdown of arginase (n = 49). NI: not injected, Arg: arginase, SF: sugar fed, BF: blood fed. Blood feeding did not mask the knockdown effect of Arg-dsRNA. Relative gene expression of arginase for each time point were calibrated against their respective LacZ injected group at the same time interval. Arg: arginase. *** = p< 0.001, ** = p< 0.01, * = p< 0.05.

The primer efficiency of all qPCR primers used in this study is provided in S2 Table. Mosquitoes injected with arginase dsRNA had a significant reduction (p<0.001) in the expression of arginase at 24-, 48-, 72- and 96-hours post-injection compared to control groups injected with LacZ dsRNA (Fig 2A). Percentage knockdown (%KD) was ≥ 80% at all time intervals considered (94.83%, 96.37%, 90.53%, and 81.21% at 24-, 48-, 72- and 96-hours, respectively) (Fig 2B). Despite the observed strong silencing of arginase, knockdown of arginase had no significant effect on the survival of the mosquitoes compared to the LacZ-injected (p = 0.39) and not injected (NI) controls (p = 0.86) for a period of 19 days post-blood meal (Fig 2C). Survival of mosquitoes did not differ significantly (p = 0.57) among the three groups. To investigate possible reasons why no effect on survival was observed despite the strong silencing, gene expression levels of arginase in mosquitoes fed with blood compared to those fed with sugar were determined (Fig 2D). Likewise, expression levels of arginase in mosquitoes fed on a blood meal 48 h after arginase or lacZ dsRNA treatment were evaluated to determine if blood feeding reverses/masks the knockdown effect of arginase dsRNA treatment earlier observed (Fig 2E). This was done because the mosquitoes in the survival experiments were given blood meal 48 h after dsRNA injection since arginase was considered a possible target for the abrogation of *P*. *berghei* development in the mosquitoes. It was observed that arginase levels were significantly reduced (p<0.01) in naïve blood-fed mosquitoes 24 h after a blood meal compared to their sugar-fed counterparts (%KD = 62%) (Fig 2D). Likewise, arginase levels were greatly reduced (p<0.001) in blood-fed arginase dsRNA-treated mosquitoes compared to their blood-fed LacZ dsRNA-treated counterparts 24 h after blood meal (Fig 2E). This showed that blood feeding did not mask or revert gene silencing. Since arginase levels are reduced during a naïve blood meal, arginase might not be essential for the survival of mosquitoes. Silencing of arginase was noted to significantly reduce (p = 0.020) the number of *Plasmodium* oocytes counts in the midgut of mosquitoes compared to the LacZ dsRNA injected control group (Fig 2F).

### Effect of knockdown of HSP, Elf2 and Elf1 on survival of *An*. *gambiae*

**Fig 3:**
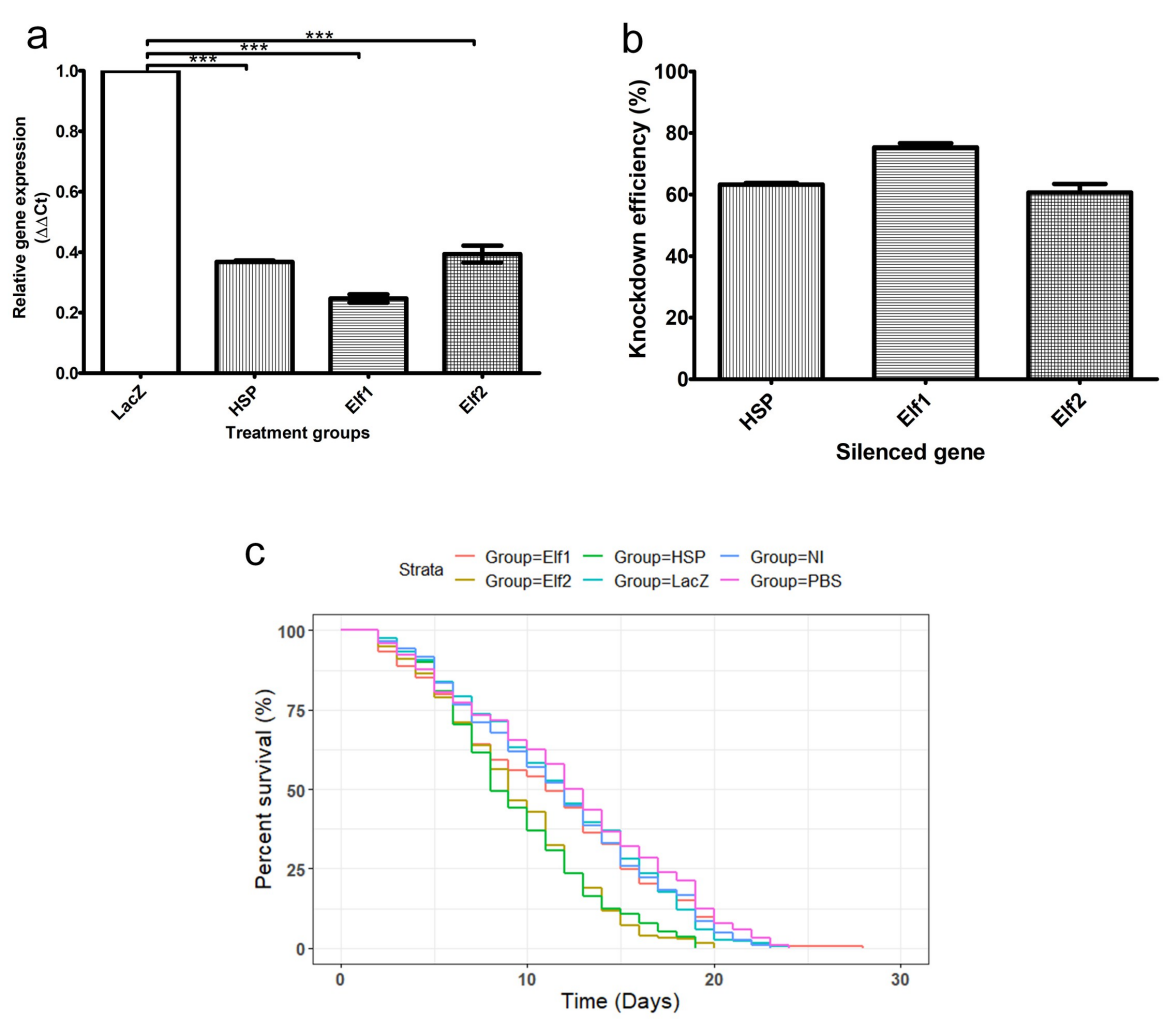
(a) Relative gene expression of HSP, Elf1, Elf2 72 h after dsRNA injection in *An*. *gambiae* G3 (n = 3) (b) Percentage knockdown of HSP, Elf1, Elf2 72 h after dsRNA injection in *An*. *gambiae* G3 (n = 3) (c) Percentage survival of female *An*. *gambiae* in NI, LacZ, PBS, HSP, Elf1 or Elf2 treated groups after knockdown. HSP: Heat shock 70kDa protein 1/8, Elf2: Elongation factor 2, Elf1: Elongation factor 1-alpha, NI: not injected, PBS: PBS injected, LacZ: LacZ injected. %KD of HSP, Elf1 and Elf2 were 63, 75 and 61%, respectively. *** = p< 0.001.

**Fig 4:**
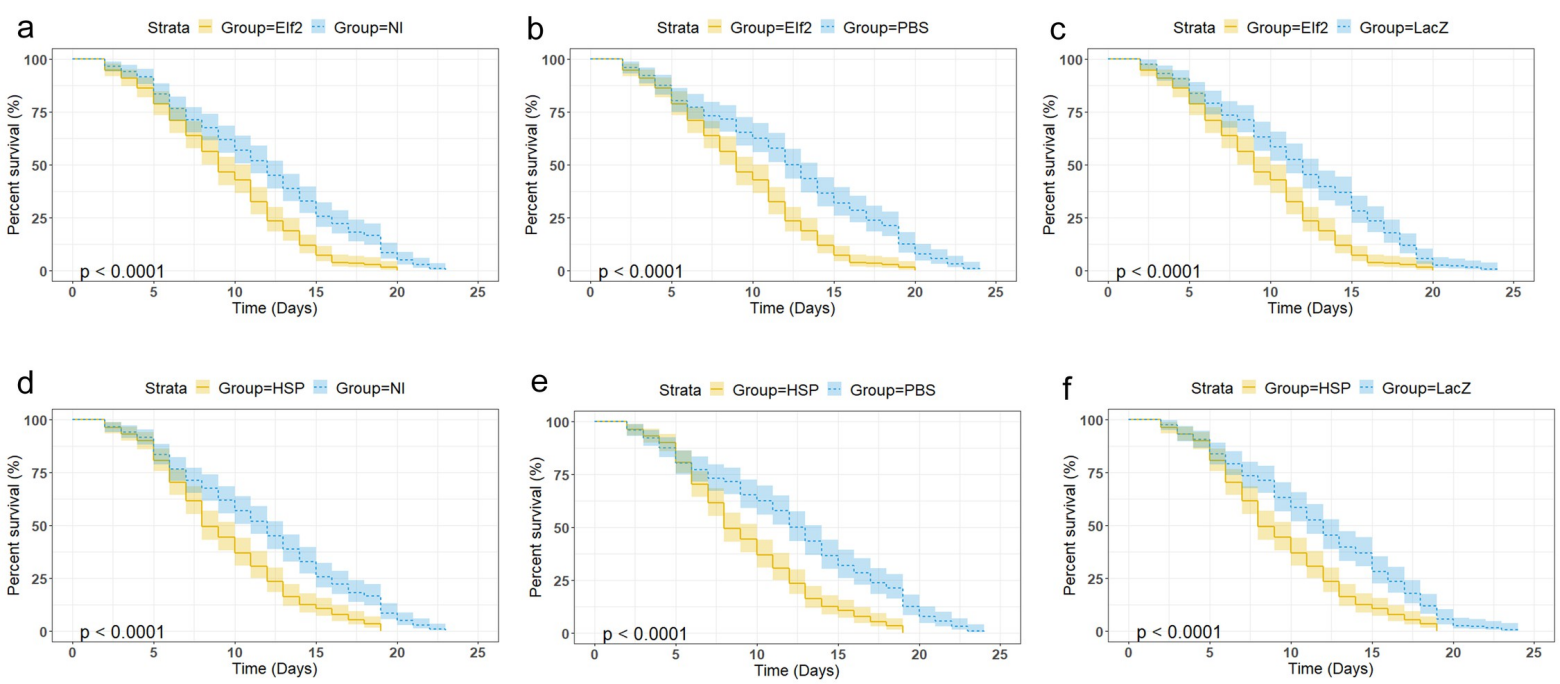
Percentage survival of female *An*. *gambiae* (a) NI compared to Elf2 dsRNA injected (b) PBS injected compared to Elf2 dsRNA injected (c) LacZ dsRNA injected compared to Elf2 dsRNA injected (d) NI compared to HSP dsRNA injected (f) LacZ dsRNA injected compared to HSP dsRNA injected (g) PBS injected compared to HSP dsRNA injected. Lines represent mean (n = 4) while bands represent confidence interval. HSP: Heat shock 70kDa protein 1/8, Elf2: Elongation factor 2, NI: Not injected, PBS: PBS injected, LacZ: LacZ injected.

*An*. *gambiae* G3 mosquitoes injected with either HSP, Elf2 or Elf1 dsRNA had a significant reduction (p<0.001) in the expression of the respective gene 72 h post-injection compared to control groups injected with LacZ dsRNA (Fig 3A). Percentage knockdown (%KD) in HSP, Elf2 and Elf1 dsRNA injected mosquitoes were 63%, 61%, and 75%, respectively (Fig 3B). Although Elf1 dsRNA injection resulted in 75% knockdown of the gene, survival of Elf1 dsRNA injected mosquitoes did not differ significantly (p>0.05) when compared to the not injected, PBS injected, LacZ injected mosquitoes (Fig 3C). However, knockdown of Elf2 or HSP significantly decreased (p<0.0001) the survival of the mosquitoes when compared to either not injected, PBS injected or LacZ dsRNA injected controls (Fig 4A–4F).

## Discussion

Arginase was considered a possible target for disrupting *P*. *berghei* development in the mosquitoes due to its observed increased expression in midgut upon *P*. *berghei* infection [29]. Longevity assay was carried out for arginase to evaluate the suitability of the gene knockdown for the *Plasmodium* infection experiment. The mosquitoes needed to survive longer, at least 10 days to allow time for *Plasmodium* infection, development, and assessment of *Plasmodium* oocytes counts. Despite the strong knockdown achieved by arginase gene-silencing, no effect on longevity of mosquitoes was noted in two longevity assay replicates (Fig 2A–2C). These two replicates did not show any variance. In both replicates, the survival of mosquitoes following knockdown of arginase was comparable to the control group. The study suggests that arginase might not be important for the survival of mosquitoes, which may be reasoned by the observation that its expression is significantly reduced during blood feeding (Fig 2D and 2E). Hence, the *Plasmodium* infection study was carried out. Similarly, no effect on survival due to arginase knockdown was observed during infection studies. In turn, knockdown of arginase resulted in a significant reduction in *P*. *berghei* oocytes counts per midgut at day 10 post-infection (Fig 2F), suggesting that knockdown of arginase hampers the development of *P*. *berghei*. Arginase competes with nitric oxide synthase for the same substrate, arginine. Parasites, e.g., trypanosomes, have been reported to evade nitric oxide production in the host by activating the production of host arginase [55]. This has been observed to result in a depletion of l-arginine, resulting in reduced levels of cytotoxic nitric oxide and enhanced production of polyamines required for parasite growth [56]. Although this complete mechanism has not been elucidated in *An*. *gambiae*, it is proposed that knockdown of arginase might result in increased abundance of nitric oxide, thereby enhancing parasite clearance [57–59]. However, this must be further investigated. In addition, arginase metabolizes arginine to produce ornithine, which is a precursor for polyamine synthesis. It has been shown that polyamines modulate *Plasmodium infection* [58, 59], hence, knockdown of arginase may prevent their synthesis thereby reducing parasite load [57]. Since the knockdown of arginase did not affect survival, it might be useful to investigate the effect of a complete knockout of arginase on the development of *Plasmodium*. Arginase shares 42.0% (E-value: 4e-87) protein sequence identity with its mitochondrial ortholog in human (P78540) and 45.4% (E-value: 3e-87) to the cytosolic human ortholog (P05089) although no significant similarity was found between their nucleotide sequences. Hence, from this study, arginase might represent a good target to explore for transmission-blocking in *An*. *gambiae*, with consideration made to developing highly selective inhibitors. The incomplete parasite clearance observed as a result of arginase knockdown could be because knockdown transiently reduces expression of genes and does not completely prevent protein synthesis, hence some arginase would be available. Likewise, immune response in mosquitoes to parasite invasion is complex, involving interaction of many proteins and cell types. The timing and intensity of these interactions can result in different outcomes [60]. Simultaneous knockdown of multiple proteins that influence these responses, resulting in enhanced parasite clearance might be necessary to achieve complete clearance.

Knockdown of Elongation factor 1-alpha, Elf1 (AGAP007406) did not affect the survival of mosquitoes despite the strong knockdown observed upon Elf1 dsRNA treatment (Fig 3A–3C). This observed result might be explained by the presence of an isoform for Elongation factor 1-alpha (AGAP003541) in *An*. *gambiae*. AGAP003541 had very low TPM values (<1) in the RNA-seq data used in this study, as compared to AGAP007406 that had TPM values of ≈8000 (see S1 File), hence AGAP007406 was considered to be the major isoform and was targeted by RNAi. Since dsRNA was designed to specifically target AGAP007406, increased expression of its isoform AGAP003541 might be triggered to perform the necessary function of the gene product, thereby counteracting the effect of silencing the AGAP007406 isoform. Elf1 is a housekeeping gene with a GTP binding protein product necessary for peptide elongation during protein translation [61], hence it is an important hub in protein networks [62]. Inhibition of eukaryotic translation elongation factor 1 alpha 1 by Nannocystin Ax has been reported to inhibit translation of new proteins and downregulate cyclin D1, inducing G1 cell cycle arrest in colon cancer cells [61]. This suggests the importance of this gene for cellular survival. To further investigate the essentiality of Elongation factor 1-alpha in mosquitoes, it would be necessary to design dsRNA that would target conserved regions between the two genes or use chemical inhibitors.

Knockdown of Elongation factor 2 (Elf2) significantly reduced the survival of *An*. *gambiae* (Figs 3C, 4A–4C). The Elf2 is a GTP-binding protein essential for protein synthesis. It catalyses the translocation of 2 tRNAs, and mRNA on the ribosome following peptidyl transfer [63]. Unlike Elf1, Elf2 is encoded by a single gene [64]. Phosphorylation of Elf2 leads to its inactivation, consequently downregulating translation and reducing peptide chain elongation [65]. Considering this crucial role of Elf2 and the uniqueness of its gene, its knockdown would result in reduced synthesis of proteins in the mosquitoes, which ultimately results in the death of the mosquito. Knockdown of Elf2 in mice downregulated expression and synthesis of proteins involved in histone and chromatin binding DNA helicase activity, while synthesis of ribosomal proteins was upregulated [64]. This suggests that Elf2 is indispensable for cell division. It has also been reported that Fragment A of Diphtheria toxin, which is produced by *Corynebacterium diphtheriae*, inhibits protein synthesis through ADP ribosylation of Elf2 (ADP ribosylation leads to inactivation). Diphtheria toxin causes diphtheria, which results in 5 to 10% death in *C*. *diphtheriae* infected patients, and the mortality rate might be up to 20% in children < 5 years or adults > 40 years [66]. Hence, inhibition of Elf2 may lead to death. While it is essential to investigate the effect of the knockdown on the transcriptome of the mosquitoes to identify the mechanism by which the observed death occurred, it is suggested that reducing levels of cell cycle proteins and other crucial proteins might contribute to the observed death. Elf2 has 78.7% (E-value: 0.0) protein sequence identity with its ortholog in human (P13639) and 79.5 gene sequence identity (E-value: 2.5e-94) to the human eukaryotic translation elongation factor 2 gene (EEF2) (NM_001961.4). Hence, caution must be taken in developing inhibitors that can be used as insecticide molecules against this target. Identifying unique and specific features in the protein of mosquitoes compared to humans can aid the development of highly specific inhibitors for this target [67]. For example, studies have shown that selective acetylcholinesterase inhibitors can be designed for *An*. *gambiae* by targeting an unpaired cysteine residue present in the mosquito but absent in humans [68, 69]. Sequence alignment of Elf2 amino acid residues from *An*. *gambiae* (AGAP009441), *An*. *stephensi* (ASTEI20_042603), *An*. *funestus* (AFUN2_002633), *Aedes aegypti* (AAEL004500), *Ae*. *albopictus* (AALFPA_058151), *Culex quinquefasciatus* (CQUJHB017554) and humans (NP_001952.1) provide evidence that selective insecticide development might be possible (S1 Fig). The sequence alignment reveals specific amino residues conserved across the mosquito species but not in human, as well as some residues conserved in the anopheline mosquitoes only. When compared to humans, there is a unique sequence region (**TNPDQRD**) present in all mosquito sequences aligned which is absent in the human sequence. Such unique residues, among others (in S1 Fig) could be exploited for the development of selective and specific insecticides that are not toxic to humans [67], once their functional role have been better studied. Hence, Elf2 might represent a good target to explore for insecticide development. Similarly, this gene could be a good candidate for RNAi-based pesticide vector control strategies.

Heat shock 70kDa protein 1/8 (HSP) significantly reduced the survival of *An*. *gambiae* (Figs 3C, 4D–4F). HSP is a molecular chaperone for protein folding, and its induction has been reported to suppress o’nyong-nyong virus (ONNV) infection. Consequently, its knockdown enhances ONNV replication in *Anopheles coluzzii* [70]. Subsequently, when ONNV was coinjected with respective dsRNA targeting HSP, survival was observed to be greatly reduced compared to control groups in which ONNV was coinjected with β-galactosidase. When dsRNA targeting HSP alone was injected in mosquitoes, reduced survival was observed, although not as high as in combination with ONVV. This shows that both the reduced HSP levels and its subsequent effect in increasing ONNV level together resulted in an increased mortality rate [70]. Hence, the finding in this study further evidences the essentiality of HSP for the survival of mosquitoes.

Further studies to evaluate the effect of HSP on *Plasmodium* development would give further insight into how HSP could be manipulated to hamper malaria transmission. HSP has also been shown to be upregulated in DDT-resistant *An*. *funetus* in Benin [71]. Similarly, AALB008255 in *An*. *albimanus*, which is identical to HSP in its carboxyl end was found to be downregulated in *P*. *berghei* infection in the mosquito [72]. There is a need to further investigate the effect of knockdown of this gene on insecticide resistance as well as *Plasmodium* infection. HSP has 80% identity with heat shock protein family A (HSP70) member 1A and 1B in human (NM_005345.6, and NM_005346.6) and 72.6% with heat shock cognate 71kDA protein isoform 1 in humans (NM_006597.6). Hence, caution should be taken if it is considered a target for insecticide development. Sequence alignment of HSP70 amino acid residues from *An*. *gambiae* (AGAP002076), *An*. *stephensi* (ASTEI20_036817), *An*. *funestus* (AFUN2_003795), *Ae*. *aegypti* (AAEL019403), *Ae*. *albopictus* (AALFPA_044680), *Culex quinquefasciatus* (CQUJHB018229) and humans (NP_006588.1 or NP_005336.3) provide evidence that selective insecticide development might be possible (S2 Fig). The sequence alignment reveals specific amino residues conserved across the mosquito species but not in human, as well as some residues conserved in these anopheline mosquitoes only. When compared to humans, there is a unique sequence region (**APGAG**) present in all mosquito sequences aligned that is absent in the human sequences. Such unique residues, among others (in S2 Fig) could be exploited for the development of selective and specific insecticides that are not toxic to humans [67].

The machine learning approach in this study is based on predicting essential genes across eukaryotes at organismal and cellular levels using CLEARER and an orthology based approach. The approach has proven to be useful for predictions of essential genes in *Tribolium castaenum*, some of which were experimentally validated to be essential [30]. The essential genes/proteins in this study could serve as probable targets for selective insecticide development, exploiting unique insect specific amino residues present in the targets compared to their human orthologs. Also, they could be targeted for RNAi biopesticide vector control strategies. In addition, these essential genes can be targeted for gene drive vector control strategies such as Cleave and rescue [73, 74], home and rescue gene drive [75].

Other studies are ongoing to develop other models that are conditionally essential. For example, a machine learning model to predict essential development stage and immune response genes in *Drosophila melanogaster* has been developed [76]. This model could be expanded to *An*. *gambiae* to predict conditionally essential genes that could be tested experimentally in the future. Still, a limitation of such prediction studies is, of course, that they typically come along with lists of predictions containing several false positives and missing false negatives. This makes it mandatory to follow up with experimental validations to reduce the false positives. Besides this, the analyses performed in this study was carried out using *An*. *gambiae* infected with *P*. *berghei*. As a future aspect, it would be intriguing observing perturbation studies in *An*. *gambiae* infected with *P*. *falciparum*.

## Conclusion

The machine learning approach was based on data of six model organisms and was applied to genome data of *An*. *gambiae* in a top-down approach. It led to new findings independent of prior expert knowledge, making it a valuable alternative to conservative ways to screen for novel targets in vector control. Of the four genes tested in this study, three were observed to be possible targets for vector control. These three genes were non-redundant. This elucidates the importance of combining computational techniques with experimental techniques in finding targets as non-redundant predicted targets might play a crucial for survival or immunity in the organism. This study provides evidence that HSP and Elf2 are important for survival of *An*. *gambiae*, as such, they could serve as possible targets for insecticide development or RNAi-based biopesticides. Similarly, knockdown of arginase was observed to reduce *P*. *berghei* oocytes count in *An*. *gambiae*, suggesting arginase as a possible transmission-blocking target in mosquitoes. As such, they could be exploited as targets for disease control.

## Supporting information

S1 Fig Sequence alignment of Elf2 amino acid residues from *An*. *gambiae* (AGAP009441), *An*. *stephensi* (ASTEI20_042603), *An*. *funestus* (AFUN2_002633), *Ae*. *aegypti* (AAEL004500), *Ae*. *albopictus* (AALFPA_058151), *Culex quinquefasciatus* (CQUJHB017554) and humans (NP_001952.1). The asterisk sign (*) indicates positions that have single and conserved amino acid residues. The full colon sign (:) indicates conservation between amino acid residues of strongly similar properties. The dot sign (.) indicates conservation between amino acid residues of weakly similar properties. Residues in red boxes are conserved across all mosquito species aligned but not in humans. Residues in blue boxes are conserved in anopheline mosquitoes only.
(TIF)

S2 Fig Sequence alignment of HSP70 amino acid residues from *An*. *gambiae* (AGAP002076), *An*. *stephensi* (ASTEI20_036817), *An*. *funestus* (AFUN2_003795), *Ae*. *aegypti* (AAEL019403), *Ae*. *albopictus* (AALFPA_044680), *Culex quinquefasciatus* (CQUJHB018229) and humans (NP_006588.1 or NP_005336.3). The asterisk sign (*) indicates positions that have single and conserved amino acid residues. The full colon sign (:) indicates conservation between amino acid residues of strongly similar properties. The dot sign (.) indicates conservation between amino acid residues of weakly similar properties. Residues in red boxes are conserved across all mosquito species aligned but not in humans. Residues in blue boxes are conserved in anopheline mosquitoes only. Residues in green boxes are conserved in *Anopheles and Aedes* mosquitoes only.
(TIF)

S1 Table Primer sequences. (DOCX)

S2 Table Primer efficiency of qPCR primers. (DOCX)

S1 File Predictions for all 10426 genes in *An*. *gambiae*. (XLSX)
