# Correction: Combination of computational techniques and RNAi reveal targets in *Anopheles gambiae* for malaria vector control

**DOI:** 10.1371/journal.pone.0321231

**Published:** 2025-03-27

**Authors:** Eunice O. Adedeji, Thomas Beder, Claudia Damiani, Alessia Cappelli, Anastasia Accoti, Sofia Tapanelli, Olubanke O. Ogunlana, Segun Fatumo, Guido Favia, Rainer Koenig, Ezekiel Adebiyi

There are errors in the author affiliations. The correct affiliations are as follows:

Eunice O. Adedeji^1,2,3,4^, Thomas Beder^5,6,7^, Claudia Damiani^3^, Alessia Cappelli^3^, Anastasia Accoti^3^, Sofia Tapanelli^8^, Olubanke O. Ogunlana^1,2,9^, Segun Fatumo^10^, Guido Favia^3^, Rainer Koenig^7^, Ezekiel Adebiyi^1,11, 12^

**1** Covenant University Bioinformatics Research (CUBRe), Covenant University, Ota, Ogun State, Nigeria, **2** Department of Biochemistry, Covenant University, Ota, Ogun State, Nigeria, **3** School of Biosciences & Veterinary Medicine, University of Camerino, Camerino, Italy, **4** Department of Biology, University of York, York, United Kingdom, **5** Medical Department II, Hematology and Oncology, University Medical Center Schleswig-Holstein, Kiel, Germany, **6** University Cancer Center Schleswig-Holstein, University Medical Center Schleswig-Holstein, Kiel and Lu¨beck, Germany, **7** Institute for Infectious Diseases and Infection Control (IIMK, RG Systemsbiology), Jena University Hospital, Jena, Germany, **8** Department of Life Sciences, Imperial College, London, United Kingdom, **9** Covenant Applied Informatics and Communication – Africa Centre of Excellence (CApIC-ACE), Ota, Ogun State, Nigeria, Uganda, **10** Department of Non-Communicable Disease Epidemiology, London School of Hygiene & Tropical Medicine, London, United Kingdom, **11** Division of Applied Bioinformatics, German Cancer Research Center (DKFZ), Heidelberg, Germany, **12** African Center of Excellence in Bioinformatics & Data Intensive Science, Makerere University, Kampala.
