# Supplementary figures and images for: Combination of computational techniques and RNAi reveal targets in Anopheles gambiae for malaria vector control
Source: PLoS One. 2024 Jul 5;19(7):e0305207. doi: 10.1371/journal.pone.0305207 (PMC11226046; doi:10.1371/journal.pone.0305207)

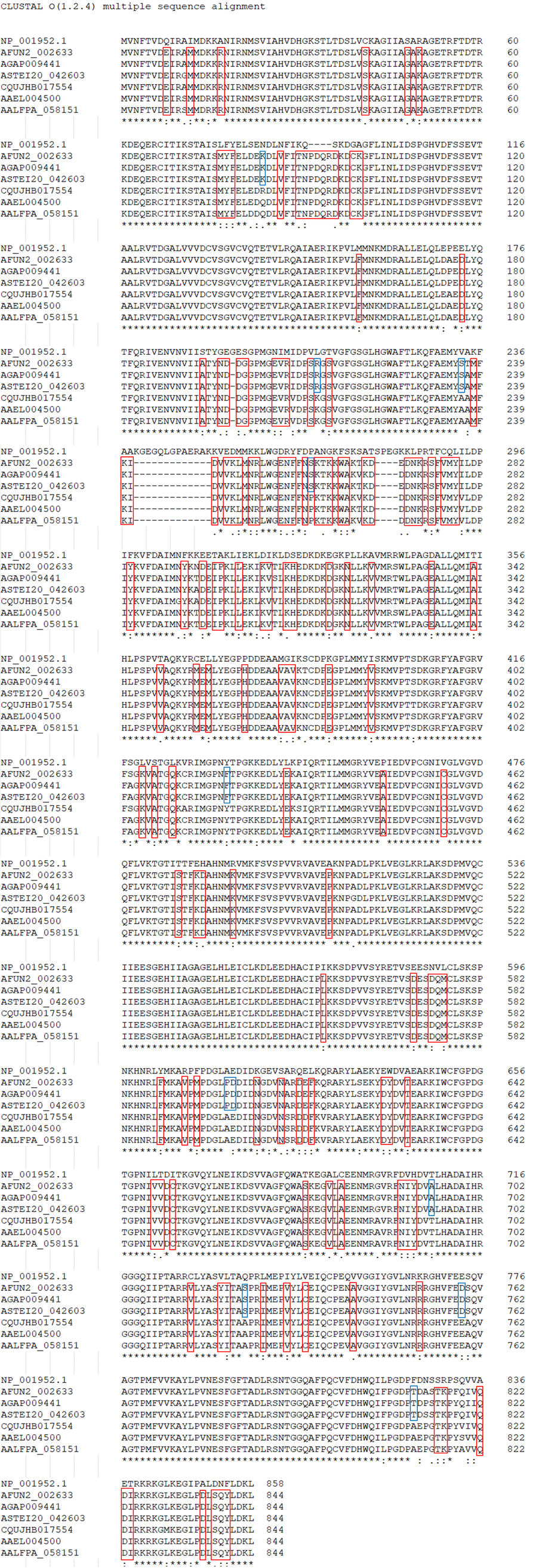

Supplement: S1 Fig — The asterisk sign (*) indicates positions that have single and conserved amino acid residues. The full colon sign (:) indicates conservation between amino acid residues of strongly similar properties. The dot sign (.) indicates conservation between amino acid residues of weakly similar properties. Residues in red boxes are conserved across all mosquito species aligned but not in humans. Residues in blue boxes are conserved in anopheline mosquitoes only. (TIF) [file pone.0305207.s001.tif]

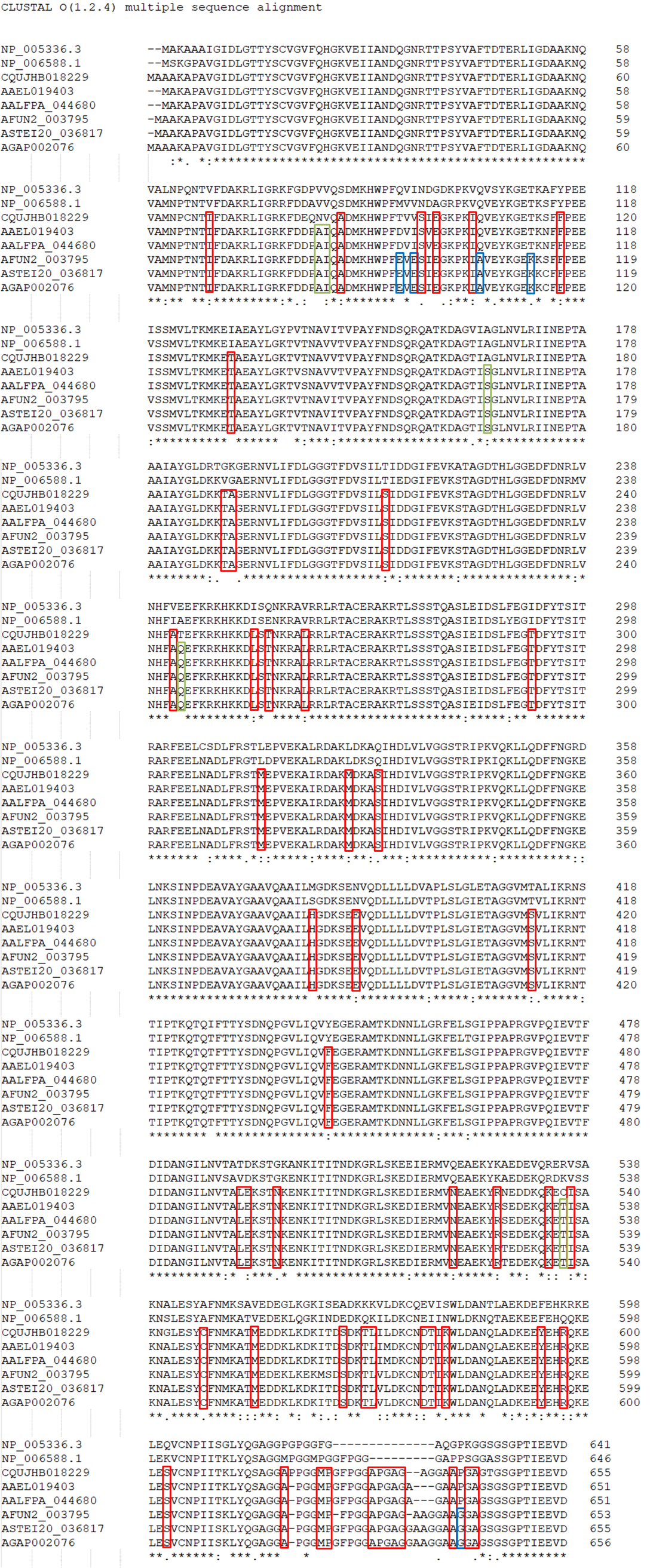

Supplement: S2 Fig — The asterisk sign (*) indicates positions that have single and conserved amino acid residues. The full colon sign (:) indicates conservation between amino acid residues of strongly similar properties. The dot sign (.) indicates conservation between amino acid residues of weakly similar properties. Residues in red boxes are conserved across all mosquito species aligned but not in humans. Residues in blue boxes are conserved in anopheline mosquitoes only. Residues in green boxes are conserved in Anopheles and Aedes mosquitoes only. (TIF) [file pone.0305207.s002.tif]
