# Supplementary material for: Combination of computational techniques and RNAi reveal targets in Anopheles gambiae for malaria vector control
Source: PLoS One. 2024 Jul 5;19(7):e0305207. doi: 10.1371/journal.pone.0305207 (PMC11226046; doi:10.1371/journal.pone.0305207)
Supplement: S1 Table — (DOCX) [file pone.0305207.s003.docx]

**S1 Table: Primer sequences**

| Purpose | Target | Primer name | Sequences |
| --- | --- | --- | --- |
| dsRNA for RNAi | Arginase (Arg)  AGAP008783 | Arg255_F | taatacgactcactatagggGTGTCACGTTTGGAGCGTC |
|  |  | Arg255_R | taatacgactcactatagggTGAGCGCTTCATACTGGATGT |
|  | Elongation factor 2 (2Elf)  AGAP009441 | 2Elf282_F | taatacgactcactatagggGCGTTCGTTTCAACATCTACG |
|  |  | 2Elf282_R | taatacgactcactatagggACTCGTTGACGGGCAGATAA |
|  | Elongation factor 1-alpha (1Elf)  AGAP007406 | 1Elf377_F | taatacgactcactatagggAGCAGTGTGTCCTCCTCCAC |
|  |  | 1Elf377_R | taatacgactcactatagggCGCAGCAGAAGAGAAGCAT |
|  | Heat shock 70kDa protein 1/8 (Hsp)  AGAP002076 | Hsp203_F | taatacgactcactatagggCTGCTTCAACATGAAGGCCA |
|  |  | Hsp203_R | taatacgactcactatagggCCTGGTACAGCTTGCTGATG |
|  | LacZ | LacZ_F | taatacgactcactatagggAGAATCCGACGGGTTGTTACT |
|  |  | LacZ_R | taatacgactcactatagggCACCACGCTCATCGATAATTT |
| qPCR | Arginase (Arg)  AGAP008783 | qArg139_F | TCGAGAAGTTTGGCATCAACGC |
|  |  | qArg139_R | AGCGAATCGATGTCGTAGCTCA |
|  | Elongation factor 2 (2Elf)  AGAP009441 | q2Elf114_F | ATCTGAGATCCAACACCGGTGG |
|  |  | q2Elf114_R | CCTGGATGATCTGGTACGGCTT |
|  | Elongation factor 1-alpha (1Elf)  AGAP007406 | q1Elf119_F | TTGCGAGCAGAGGATACACACA |
|  |  | q1Elf119_R | CGCATCCTCTACTCCTTTCCCT |
|  | Heat shock 70kDa protein 1/8 (Hsp)  AGAP002076 | qHsp112_F | GCGATCGTTGTGTGAGAGTGTG |
|  |  | qHsp112_R | GAAGTCCCGAGAAGCGCAAAAT |
|  | Ribosomal protein S7  AGAP010592 | qS7112_F | CATTCTGCCCAAACCGATGCG |
|  |  | qS7112_R | CGGGAATACCAGATCCTCCAGG |
