# Supplementary material for: Combination of computational techniques and RNAi reveal targets in Anopheles gambiae for malaria vector control
Source: PLoS One. 2024 Jul 5;19(7):e0305207. doi: 10.1371/journal.pone.0305207 (PMC11226046; doi:10.1371/journal.pone.0305207)
Supplement: S2 Table — (DOCX) [file pone.0305207.s004.docx]

**S2 Table: Primer efficiency of qPCR primers**

| Gene | % Efficiency | Slope | R^2^ | Efficiency (E) |
| --- | --- | --- | --- | --- |
| S7 | 98.7 | -3.353 | 0.982 | 1.987194524 |
| Tre | 100.6 | -3.307 | 0.998 | 2.006267658 |
| Arg | 94 | -3.475 | 0.992 | 1.939857303 |
| HSP | 96.7 | -3.404 | 0.999 | 1.96685359 |
| 1Elf | 103.6 | -3.239 | 0.997 | 2.0358101 |
| 2Elf | 95.6 | -3.431 | 0.999 | 1.956411539 |
